# Antimutagenic and antioxidant activity of the essential oils of *Citrus sinensis* and *Citrus latifolia*

**DOI:** 10.1038/s41598-017-11818-5

**Published:** 2017-09-13

**Authors:** J. D. Toscano-Garibay, M. Arriaga-Alba, J. Sánchez-Navarrete, M. Mendoza-García, J. J. Flores-Estrada, M. A. Moreno-Eutimio, J. J. Espinosa-Aguirre, M. González-Ávila, N. J. Ruiz-Pérez

**Affiliations:** 1 Laboratorio de Investigación Microbiológica, UIMyT, Hospital Juárez de México, Av. Instituto Politécnico Nacional # 5160 Col. Magdalena de las Salinas, Ciudad de México, México, C.P. 07650 Mexico; 2 Laboratorio 5, UIININ, Hospital Juárez de México, Av. Instituto Politécnico Nacional #5160 Col. Magdalena de las Salinas, Ciudad de México, México, C.P. 07650 Mexico; 3 Laboratorio 7, UIININ, Hospital Juárez de México, Av. Instituto Politécnico Nacional #5160 Col. Magdalena de las Salinas, Ciudad de México, México, C.P. 07650 Mexico; 4 Departamento de Medicina Genómica y Toxicología Ambiental. Instituto de Investigaciones Biomédicas Edificio C, 2° Piso, Lab C-205. Circuito Mario de la Cueva s/n, Coyoacán, Ciudad Universitaria, 04510 Ciudad de México, Mexico; 5 Centro de Investigación y Asistencia en Tecnología y Diseño del Estado de Jalisco A.C. Av. Normalistas # 800 Col. Colinas de la Normal, Guadalajara, Jal. México, C.P. 44270 Mexico

## Abstract

The essential oils of *Citrus sinensis* and *Citrus latifolia* showed antimycotic activity against *Candida spp*. isolated from the oral cavity; they are neither mutagenic on the Ames test nor cytotoxic. Their main components are R-(+)-limonene, β-thujene, α-myrcene and γ-terpinene. The aim of this work was to evaluate their antimutagenic and antioxidant capacities. Antimutagenic properties were evaluated against MNNG and ENNG on *S. typhimurium* TA100; against 2AA on strain TA98 and in front of 4NQO and NOR on strain TA102. Both were antimutagenic against MNNG (p < 0.001) but only *C. latifolia* was antimutagenic against ENNG (p < 0.001). Both presented antimutagenic activity against 2AA (p < 0.001). They were antioxidant against the ROS-generating compound 4NQO (p < 0.001) and the antibiotic NOR (p < 0.001). In the antioxidant evaluation, the activity in DPPH assay was in a range of 6–23% for *C. sinensis* and of 22–71% for *C. latifolia*. Both were antioxidant compared with BHT in β-carotene bleaching assay and were able to decreased apoptosis in HaCat cells stimulated with H_2_O_2_. The levels of intracellular superoxide ion were lower in the presence of both oils. In conclusion, the essential oils of *C. sinensis* and *C. latifolia* are antimutagenic against at least three types of mutagens and have antioxidants properties.

## Introduction

Essential oils (EOs) of natural origin are volatile liquid fractions, generally, steam-distillable containing substances responsible for the aroma of plants. EOs are important ingredients in the cosmetic industry, in food as flavorings or condiments and in the pharmaceutical production^1^. In particular, the *Citrus* genus is a source of vitamin C, flavonoids and terpenoids that have been the subject of study due to their demonstrated beneficial properties. The whole oils and its different components have been recognized as generally safe (GRAS) by the Food and Drug Administration (FDA)^2^.

We previously demonstrated that the EOs of *Citrus sinensis* and *Citrus latifolia* are antimycotic against *Candida albicans, Candida tropicalis, Candida guilliermondii, Candida glabrata* and *Candida lusitaniae* strains isolated from the oral cavity of elder patients. Both EOs were not mutagenic in the Ames test and not cytotoxic to the human oral epithelium. Based on these results, we are extending the analysis on their properties by testing its antimutagenic and antioxidant activities^3^.

The main components of these EOs were R-(+)-limonene and α-myrcene for *C. sinensis*, and R-(+)-limonene, β-thujene and γ-terpinene for *C. latifolia*, these molecules may have further biological activities. Antonella *et al*. 2013 showed that the R-(+)-limonene, α-terpineol and its chemical derivative 1,8-cineol^4^, were able to inhibit mutagenesis induced by 2-amino-anthracene, 2-amino-fluorene and alkylating agent Methyl-metano-sulfonate on *S. typhimurium* TA98, TA100 and *E. coli* uvrA strains^5^. R-(+)-Limonene and β-pinene, have been described as the bioactive components of several herbs and spices used in food preparation^6^. The concentration and proportion of these components are variable, but several epidemiological reports indicate that their consumption may reduce gastric cancer risk^6^.

Thujene, contained in these EOs has shown to be a good antioxidant, able to efficiently quenching singlet oxygen^7^. The monoterpenes such as R-(+)-limonene, γ-terpinene and β-pinene were identified as good antioxidants by the DPPH method^8^.

R-(+)-Limonene and perillyl alcohol have been described as dietary monoterpenes that may inhibit post-transcriptional isoprenylation of cell growth regulatory proteins such as ras^9^. The use of these antioxidants and antimutagens in cancer prevention has been widely evaluated, and it has been reported that these dietary monoterpenes have chemotherapeutic activity in pancreatic, mammary and prostatic tumours^10^. Another example of chemotherapeutic compounds is the juice of *Citrus medica*, which supplementation was antimutagenic and anticarcinogenic in human astrocytoma cell lines^11^.

Several studies have been developed with whole essential oils of several plants. Bhalla *et al*. (2013) reported that antimutagenesis of EOs might be due to antioxidant quenching reaction, induction of detoxification enzymes and inhibition of CYP450 involved in activation of pre-carcinogens. The antimutagenesis of EOs involving DNA repairing mechanisms is perhaps the less studied process^12^. Other study had shown that some EOs might be implicated with DNA repairing systems. The EO of basil (*Ocimun basilicum L*.) protects *Escherichia coli* from ultraviolet light-induced mutagenesis, only on a DNA excision repairing proficient strain^13^. In fact, there are other works that had stated that DNA repairing improvement mechanisms by essential oils and extracts would have to do with the antimutagenic mechanisms^14^. Nonetheless, the antioxidant capability of natural compounds is perhaps the action mechanism most studied and documented in the literature due to the importance of oxidative reactions on cell damage^15^.

One method that is a standard to evaluate genotoxic risk is the Ames test, which is accepted by multiple organizations (IARC, EPA, FDA, IVGT, REAC, MHW-Japan) as a screening test of compounds for human consumption and use. It is based on strains harboring a series of mutations that are reverted in the presence of a mutagen; each strain used in this methodology allows the identification of mutations by frameshifting (TA98), base substitution (TA100) or damage by free radicals (TA102). Additionally, it has been used to prove the antimutagenic activity of compounds from diverse origins in front of a known mutagen, by measuring the decrease in the number of revertants (His+). It is an approach to elucidate the mechanism of action of both mutagenic and antimutagenic molecules^16^.

The aim of this work was to further evaluate the properties of essential oils from *Citrus sinensis* and *Citrus latifolia* such as antimutagenic and antioxidant activities.

## Results

### Antimutagenesis

Antimutagenic effects frequently are dependent on the mutagen and antimutagen doses used. The doses of EOs used in this work were not toxic for the strains, when evaluated alone.

**Figure 1:**
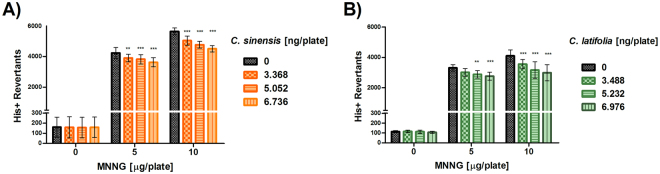
Antimutagenesis in *S. typhimurium* TA100. (**A**) Antimutagenesis of *Citrus sinensis* against mutations induced by MNNG. Spontaneous reversion 161 ± 80 **p < 0.01, ***p < 0.001. (**B**) Antimutagenesis of *Citrus latifolia* against mutations induced by MNNG. Spontaneous reversion: 114 ± 10, **p < 0.01, ***p < 0.001. Each point represents the mean of 15 replicas on five independent studies.

Both essential oils were able to inhibit alkylating mutations induced by MNNG. *C. sinensis* (Fig. 1A) and *C. latifolia* (Fig. 1B) were antimutagenic at all its doses, even when the strain was exposed to 10 μg of MNNG (p < 0.001). Nonetheless, the EO of *C. latifolia* had not activity in front of 5 µg/pl of MNNG when applied at its lowest concentration.

**Figure 2:**
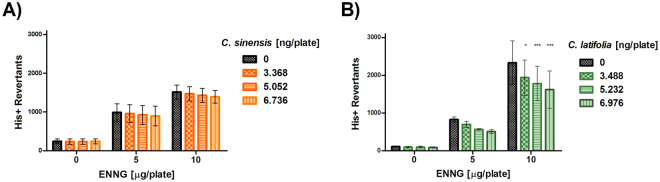
Antimutagenesis in *S. typhimurium* TA100. (**A**) Antimutagenesis of *Citrus sinensis* against mutation induced by ENNG. Spontaneous reversion 239 ± 10. (**B**) Antimutagenesis of *Citrus latifolia*, against mutation induced by ENNG. Spontaneous reversion: 239 ± 10. Each point represents the mean of 15 replica on five independent studies, *p < 0.05, ***p < 0.001.

For the ethylating agent ENNG, there was not a significant reduction in the number of His+ revertants at neither *C. sinensis* doses used in front of 5 and 10 µg/plate of this alkylating agent (Fig. 2A). A gradual reduction in the number of His+ revertants was observed with increasing amounts of *C. latifolia* against 10 µg/plate of ENNG (p < 0.05 for 3.488 ng/pl; p < 0.001 for 5.232 and 6.976 ng/pl). However, when the TA100 strain was exposed to 5 µg/pl of ENNG there was not a difference with and without the EO of *C. latifolia* (Fig. 2B).

**Figure 3:**
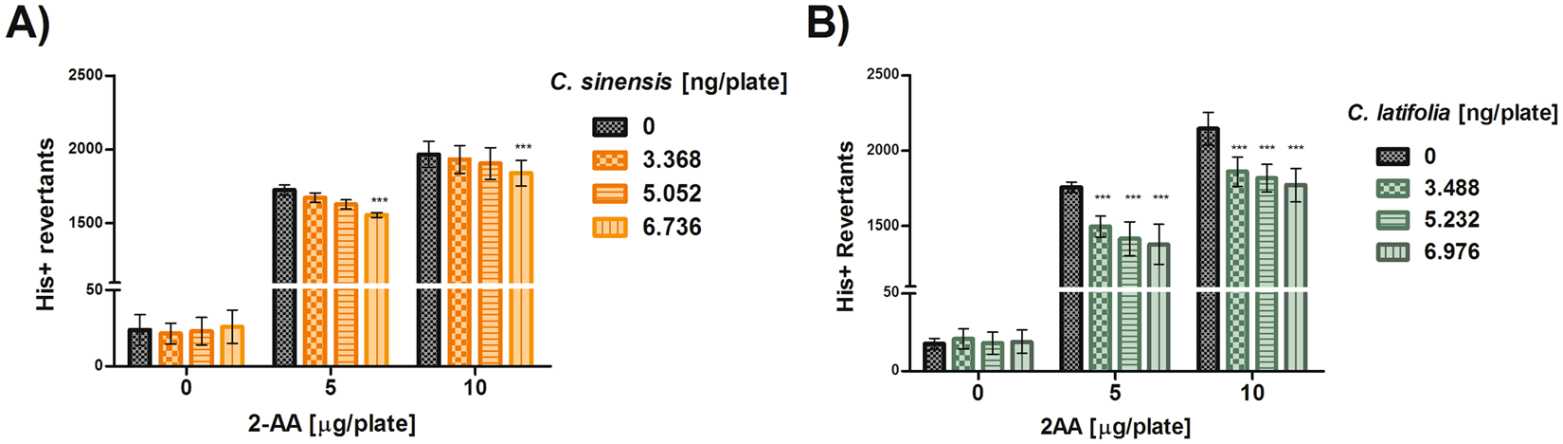
Antimutagenesis in *S. typhimurium* TA98. (**A**) Antimutagenesis of *Citrus sinensis* against mutation induced by 2AA. Spontaneous reversion 16 ± 3. ***p < 0.001. (**B**) Antimutagenesis of *Citrus latifolia*. Spontaneous reversion 17 ± 5, ***p < 0.001. Each point represents the mean of 15 replicas on five independent studies.

Antimutagenesis against 2AA was significant only at the highest concentration of *C. sinensis* against both doses of this pre-mutagen (p < 0.001) (Fig. 3A). In contrast, *C. latifolia* presented an evident antimutagenic activity at all its concentration (p < 0.001) and with both concentrations of 2AA (Fig. 3B).

**Figure 4:**
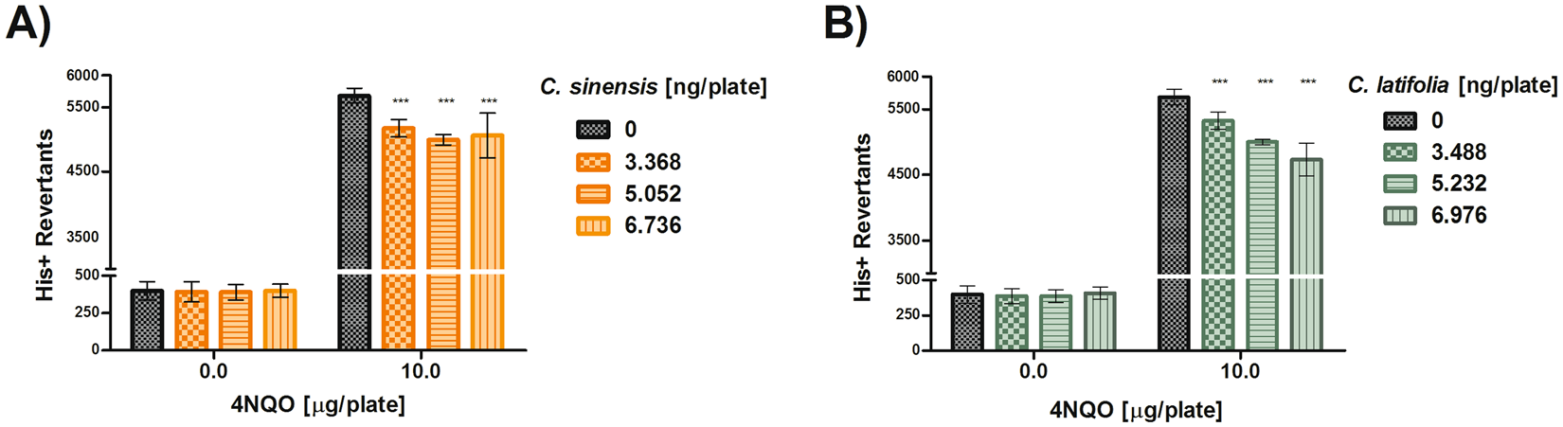
Antimutagenesis in *S. typhimurium* TA102. (**A**) Antimutagenesis of *Citrus sinensis*, against 4-NQO. Spontaneous reversion 266 ± 30 ***p < 0.001. (**B**) Antimutagenesis of *Citrus latifolia*, against 4NQO. Spontaneous reversion 266 ± 30 ***p < 0.001. Each point represents the mean of 9 replicas on three independent studies.

Mutations induced by 4NQO were reduced with both EOs (Fig. 4). *C. sinensis* (Fig. 4A) or *C. latifolia* (Fig. 4B) had a statistically significant antimutagenic effect at every proved concentration (p < 0.001).

**Figure 5:**
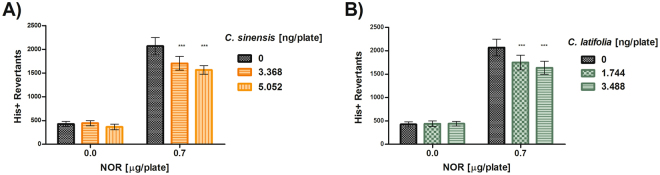
Antimutagenesis in *S. typhimurium* TA102. (**A**) Antimutagenesis of *Citrus sinensis*, against NOR. Spontaneous reversion 412 ± 23 ***p < 0.001. (**B**) Antimutagenesis of *Citrus latifolia*, with NOR. Spontaneous reversion 412 ± 23 ***p < 0.001. Each point represents the mean of 9 replicas on three independent studies.

The antioxidant effect of these essential oils was also evaluated against NOR (Fig. 5A and B). Both EOs shown a dose-dependent and statistically significant reduction of mutations induced by this ROS-generating antibiotic.

### Antioxidation

#### DPPH free radical scavenging

**Figure 6:**
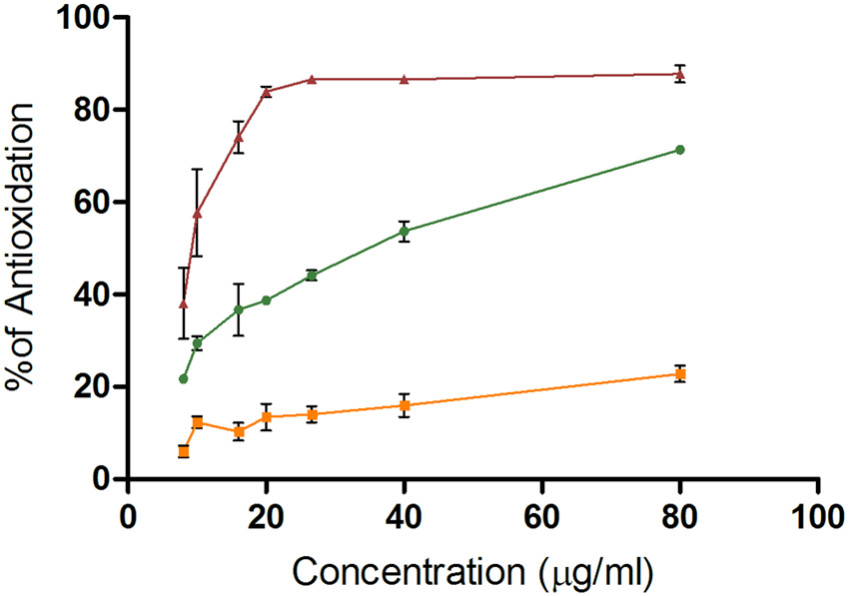
Graphic of the % of antioxidation *versus* concentration of  EGCG,  *C. sinensis* and  *C. latifolia*.

To ratify whether the results on the TA102 strain were related to the antioxidant activity of these EOs, we performed a DPPH assay compared with EGCG, a flavonoid with reported antioxidant activity from green tea. The Figure 6 shows the percentage of the antioxidation of the EGCG (38–88%), *C. latifolia* (22–71%) and *C. sinensis* (6–23%). It could be observed that the EO of *C. latifolia* had a better activity than the EO of *C. sinensis*, although both were lower than EGCG.

#### Bleaching of β-carotene in linoleic acid system

**Figure 7:**
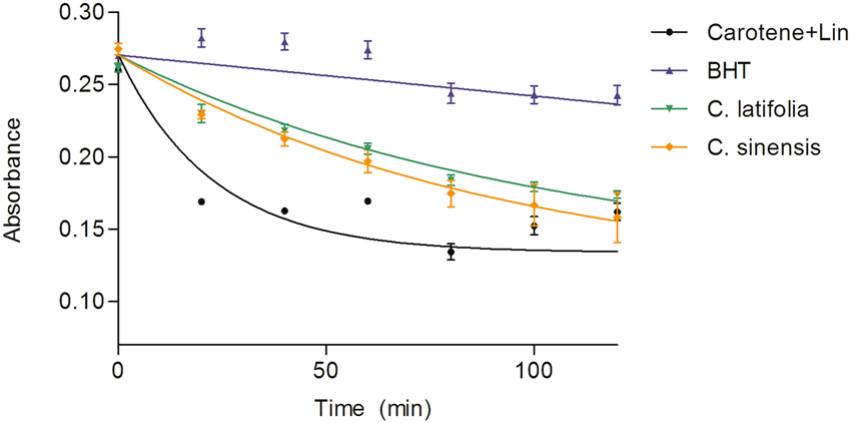
Effect of the EOs in the Inhibition of β-carotene bleaching.  Control (25 µL Linoleic acid + 0.8 mg β-carotene),  BHT (400 mg),  *C. sinensis* (400 mg) and *C. latifolia* (400 mg). Each point is the mean ± S.D. of three biological experiments performed by triplicate.

The antioxidant activity of the essential oils of *Citrus sinesis* and *Citrus latifolia* by bleaching of β-carotene with the linoleic acid is shown in Fig. 7. The more effective an antioxidant the slower the depletion of color. The increase in the absorbance of β-carotene indicates less oxidation by linoleic acid. The control BHT showed a lower exhaustion of color than the essential oils. Based on this, the results were BHT > *C. latifolia* > *C. sinensis*.

#### Detection of apoptosis using flow cytometry

**Figure 8:**
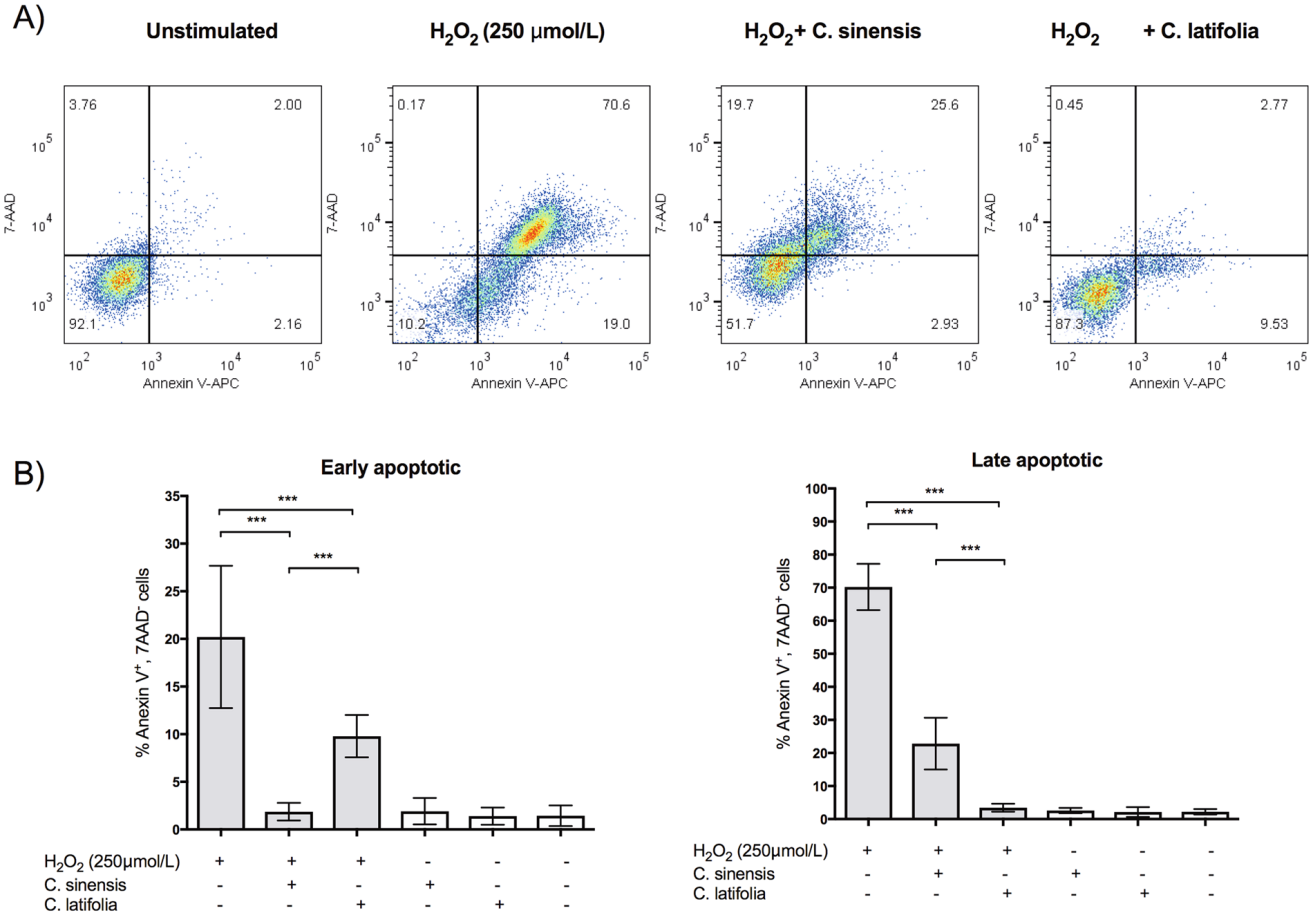
Cell apoptosis measured by flow cytometry. (**A**) Representative density plots in the presence and absence of the EOs. (**B**) Quantization of early and late apoptosis as % Annexin V-7AAD positive cells. Results are presented as mean ± S.D. ***p < 0.0001.

To demonstrate the antioxidant activity of the EOs *in vivo*, the number of cells in early and late apoptosis was measured by flow cytometry using Annexin V staining and 7-ADD as cell membrane integrity exclusion dye. Figure 8A shows representative density plots where *C. latifolia* maintains the cell integrity in presence of H_2_O_2_ in a higher proportion (87.3% of survival) than *C. sinensis* (51.7% of survival), compared with damage cells (10.2% of survival). Figure 8B illustrate the quantitation of early and late apoptosis with and without the EOs. *C. sinensis* reduces the late apoptosis from 70.2% to 22.8% and *C. latifolia* from 70.2% to 3.4%. Meanwhile the early apoptosis was reduced from 20.2% to 1.84% and 9.79%, respectively.

#### Measurement of intracellular reactive oxygen species

**Figure 9:**
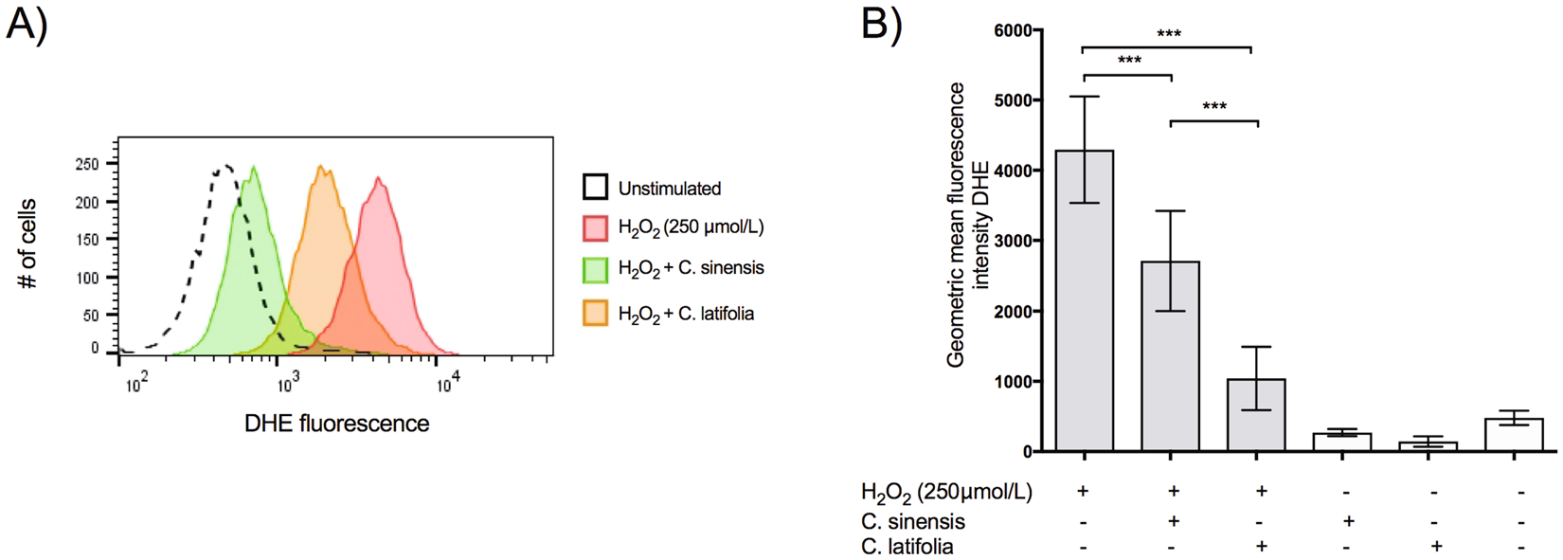
Inhibition of intracellular superoxide formation. (**A**) Histogram of the ethidium fluorescence with and without EOs. (**B**) Quantization of the mean intensity in treated and untreated cells. ***p < 0.0001.

To test the formation of ROS inside the cells, an analysis was performed by flow cytometry. Dihydroethidium (DHE) was used to detect intracellular •O^2−^. Given its ability to freely permeate cell membranes, DHE has extensively been used to monitor •O^2−^ production. Upon reaction with •O^2−^, DHE is rapidly oxidized to form ethidium, a red fluorescent product that intercalates DNA and amplifies the red fluorescence signal. Figure 9A shows the decrease in the signal of DHE within HaCaT cells in the presence of the EOs of *C. sinensis* (orange curve) and *C. latifolia* (green curve), compared with cultures stimulated only with H_2_O_2_ (red curve). Cells treated with the EOs were significant different from untreated cells (9B, p < 0.0001).

## Discussion

Essential oils are a mixture of volatile compounds, generally classified in two major biosynthetic groups: terpenes/terpenoids and aromatic/aliphatic molecules^17^. In a precedent work, we found that EOs from *C. sinensis* and *C. latifolia* are antimycotic and besides are neither mutagenic nor cytotoxic to human oral epithelium^3^. Added up to these qualities, in here we demonstrated that these EOs also have antimutagenic properties. The use of antimutagens is a good alternative to reduce genotoxic risk to the exposure to xenobiotic compounds, and to therapeutic drugs^18^. These studies are worthwhile as they may offer an alternative for the prevention of genetic damage and even more to reduce carcinogenic risks^19^.

The reduction of mutagenesis induced by methyl or ethyl N-nitroso guanidines using the essential salmonella battery of the Ames test, suggests that these essential oils might have an antimutagenic effect against alkylating agents. The *Citrus* genus has previously been studied that reduce DNA damage by alkylating mutagens like methyl-methane-sulfonate (MMS), cyclophosphamide (CP) and ifosfamide. Orange juice prevented and repaired the damage induced by MMS and CP in comet assay and the grapefruit juice reduced the micronucleus formation and sister chromatid exchanges *in vivo* ^20–22^. The antimutagenic properties of the EOs of *C. sinensis* and *C. latifolia* observed in the present work are in accordance with the aforementioned studies on the antimutagenic effect against alkylating agents.

Our results demonstrated a slight reduction of the mutagenic effect of the pre-mutagen 2AA, which requires to be activated mainly by CYP1A1 (present on the S9 mix used in here) to be metabolized into a mutagenic product^23^. *C. sinensis* was only anti-mutagenic at a dose of 6.736 ng/plate with exposures of 5 and 10 μg/plate of 2AA. The EO of *C. latifolia* prevented the mutagenic transformation of 2AA at all concentrations. This result is in accordance with previous reports founding that grapefruit juice, which contains organic components like bergamottin, is able to reduce mutagenesis of pre-mutagen genotoxic compounds such as benzo(a)pyrene in the Ames test^24^. The consumption of *C. sinensis* and *C. latifolia* may be very useful as protection against the mutagenic effect of pre-mutagens polycyclic aromatic compounds. Nevertheless, antimutagens that are able to inhibit the cytochrome activity should be taken carefully. It has been discussed that they may also inhibit other members necessary to metabolize clinical drugs, and interfere with its therapeutic metabolism^25, 26^. Further studies in the application of these antimutagens should be advisable.

The main components of the tested citrus EOs are terpenes/terpenoids, as we previously analyzed by GC-MS^3^, which have been attributed with antioxidant properties. In fact, there are several references reporting antioxidant properties of medical plants. For instance, Bouchekrit *et al*. (2016) found that the EO of *Elaeoselinum asclepium* has a 43.9% of α-pinene and has not only antibacterial but antioxidant properties^27^. *Castella texana*, an antiamoebic plant, also presented antioxidant properties through a reduction in the genotoxic DNA damage induced by ROS, generated by fluoroquinolone NOR^28^. In regard to the citrus genus, Loizzo *et al*.^8^, reported antioxidant effects of the essential oil of *Citrus x limon* cv Femminello comune, which contains limonene, γ-terpinene and β-pinene as its major components.

In this work we demonstrated the antioxidant properties of our essential oils, although the plotted curves where lower than the reference compound (EGCG, 38–87%). It is worth mentioning that EGCG is a flavonoid from green tea considered as a potent antioxidant^29^ hence the effect of *C. sinensis* and *C. latifolia* (6–23% and 22–71%, respectively) is not despicable, especially for the latter. This effect has been studied more frequently in *C. sinensis* than *C. latifolia* although some were performed using oils or extracts obtained in different conditions^30–32^.

These results were corroborated using a lipophilic method (β-carotene bleaching) which proved that the essential oil of *C. latifolia* is more potent antioxidant than *C. sinensis* suggesting that the effect depends on the composition of each oil. *C. sinensis* is essentially R-(+)-limonene (96%), although the EO of *C. latifolia* has a lower concentration of this terpene, its second most abundant component, β-thujene (14.85%), absent in *C. sinensis*, might be contributing to the observed antioxidant activity. On the other hand, the annexin V/7AAD assays showed that the EO of *C. sinensis* decreased the early apoptosis while *C. latifolia* reduced the late apoptosis. In fact the essential oil of *C. latifolia* is able to prevent the total apoptosis in a higher percentage (85% of viable cells) than *C. sinensis*, which has not been previously described.

Other works had measured the antioxidant activity of some components of the *Citrus* genus (limonene and others) and had demonstrated that are able to protect epithelial cells from oxidative stress, which partially explains the effect of our EOs.

The measurement of the intracellular superoxide ion corroborated that the EOs act like free radical scavengers since the fluorescence intensity is lower when added to the cells, which could explains its effect in apoptosis.

All together, these results suggest that there is a possible contribution (synergic or additive) of the components present in *C. latifolia* with the R-(+)-limonene in the antioxidant activity of this essential oil.

Antioxidation is perhaps the most frequently evaluated mechanism of action for natural occurring molecules, and it might be part of the antimutagenic capacity of our EOs, based on the observations made on TA102 strain, HaCaT cells and for the formation of intracellular superoxide.

In comparison with other species, previous studies have established an antimutagenic activity for the essential oil of *Myrtus communis*, a small evergreen shrub from northwestern Europe belonging to the same taxonomic division, class and subclass than *Citrus* genus. *M. communis* contains a terpenes/terpenoids composition^3, 33^ similar to *C. sinensis* and *C. latifolia*, suggesting that some of the shared molecules could be attributed with this observed quality. We hypothesized that the β-pinene could be also an antimutagenic component since its abundance in each oil seems to concur with their antimutagenic potency (0.358% in *C. sinensis* y 12.79% in *C. latifolia* ^3^). The γ-terpinene is another homologous component in *M. communis* that is relatively abundant (12.8%), although is present only in the essential oil of *C. latifolia*. Finally, α-pinene, myrcene, and α-thujene are also compounds in common, nonetheless they were found in minimal abundance in our EOs. In contrast, the abundance of R-(+)-limonene, the most concentrated element in our citrus oils, is inversely proportional to their activity, suggesting that there is a limited participation of R-(+)-limonene in the antimutagenic, antioxidant and antifungal activities^3^ of *C. sinensis* and *C. latifolia*. Although, more studies on the capacities of each component of these essential oils are necessary to reach a more accurate conclusion.

## Conclusions

The evaluated EOs from *C. sinensis* and *C. latifolia*, presented in this work can act by several antimutagenic mechanisms; they are able to reduce alkylated DNA damages through a reduction in the expression of base-substitution mutations; also, both are antimutagenic by reducing the activation of pre-mutagens like 2AA, and, finally, against quinolones (NOR and 4NQO) as possible ROS-scavenging mixtures as proven by DPPH, β-carotene bleaching and oxidative stress assays.

## Methods

### Reagents

Essential Oils from *C. sinensis* and *C. latifolia* were obtained by hydro-distillation of the peel, and were kindly donated by Frutech International Corporation Cargee Additives, Montemorelos Nuevo León, México. Methyl-N ´nitro-N-nitrosoguanidine (MNNG), Ethyl-N-nitro-N-nitrosoguanidine (ENNG), 2-amino-anthracene (2AA), 4-nitro-quinone-oxide (4NQO), and norfloxacin (NOR) were purchased from Sigma Chemical Co. (St. Louis, MO, USA). Dimethyl sulfoxide (DMSO) was obtained from J.T. Baker Xalostoc, México. Aroclor-1254 was obtained from Supelco Bellefonte, PA; S9 arochlor-1254 induced rat liver homogenate (S9 mix), was prepared as described by Maron and Ames^29^. Diphenyl-2-picrylhydrazyl (DPPH) and the reference standards Epigallocatchin-3-gallate (EGCG) were purchased from Sigma Chemical Co. (St. Louis, MO, USA). β-carotene was kindly donated by Dra. Rosa Martha Pérez-Gutiérrez. Chloroform and Linoleic acid were purchased from Sigma-Aldrich (St. Louis, MO, USA). RPMI-1640 was purchased from Life Technologies (TermoFisher, Waltham, MA, USA). Annexin V-Allophycocyanin (APC) and 7-amino-actinomycin D (7-AAD) Apoptosis Detection kit (Biolegend, USA) was provided by Dr. Mario Adán Moreno-Eutimio. DHE was purchased from Sigma-Aldrich (St. Louis, MO, USA).

### Cell culture

HaCaT normal human epidermal keratinocyte cell line were cultured in RPMI-1640 supplemented with 10% Fetal Bovine Serum (FBS) and 1% penicillin-streptomycin (Hyclone; GE Healthcare Life Sciences, Logan, UT, USA) at 37 °C in an atmosphere containing 5% CO_2_.

### Antimutagenesis Assays

Antimutagenesis assays were performed with the Ames test on *Salmonella typhimurium* ^29^. Briefly, overnight cultures of *S. typhimurium* TA98, TA100 or TA102 strains were prepared on nutrient broth Oxoid No. 2 for 16 hrs at 37°C onto a water bath (American Scientific Products BT-23). Antimutagenic activity of both EOs was evaluated against 2AA (5 or 10 μg/Petri dish) on *S. typhimurium* TA98; MNNG and ENNG (5 or 10 μg/Petri dish) on TA100; against 4NQO (5 or 10 μg/Petri dish) on TA102. 100 μL of the exposed tester strain, both mutagens and essential oils were poured on sterile tubes containing 2.0 mL of soft-agar at 45 °C; 1.68 to 8.2 ng /Petri dish of *C. sinensis* and 1.74 to 6.97 ng/Petri dish of *C. latifolia* were used, these concentrations were previously described as non-toxic for the Ames test^1^. 2AA was activated with 500 μL of S9 mix, the mixture was then poured onto Vogel-Bonner plates and incubated for 48 hrs. Antimutagenesis of essential oils against NOR was evaluated with the pre-incubation method, previously described^16, 29^. EOs, NOR (0.07 to 7.0 ng) and 500 μL (S9 mix) were poured onto an sterile tube along with 100 μL of the tester strain and then incubated at 37 °C for 60 min, afterwards 2.0 mL of soft agar were added into the mixture and finally poured onto Vogel-Bonner Petri dishes for a 48 hrs incubation. Blanks were the strains with 10 μL of DMSO, and negative controls were all EOs doses without mutagen. Positive controls were the appropriate mutagen without the EOs^12^.

### Antioxidant activity

#### DPPH free radical-scavenging assay

Antioxidant activity of the EOs was determined through the production of DPPH radicals. Briefly, reaction mixtures of each oil were prepared with 200 µM DPPH in ethanol in a final volume of 180 µL. Serial dilutions of the EOs of *C. sinensis* or *C.latifolia* (80, 40, 26.66, 20, 16, 10 and 8 µg/mL) were measured. Epigallocatchin-3-gallate (EGCG) was used as a reference antioxidant at the same concentration that the EOs. After incubation at room temperature for 30 min, the absorbance was acquired at 517 nm using a microplate reader (Eon™Microplate Spectrophotometer, bioTek, VT, USA). The results were calculated as inhibition percentage of DPPH radical formation (% of antioxidation).

#### Bleaching of β-carotene in linoleic acid system

The antioxidant activity of the essential oils of *C. latifolia* and *C. sinensis* was also evaluated by β-carotene/linoleic acid bleaching following the recommendations of Prieto *et al*.^34^ and as previously reported in Koleva *et al*.^35^. Briefly, a stock was prepared of an emulsion of β-carotene-linoleic acid by dissolving β-carotene at 0.8 mg/ml in chloroform, 25 μl of linoleic acid and 200 mg Tween 40. The chloroform was removed under vacuum on a centrifuge (V-AL; vacofuge plus; eppendorf) at 200 x g. The content of each tube was emulsified in 50 ml of mili-Q water saturated with oxygen (30 min, 18.2 mΩ) with vigorous stirring. 1.0 ml of this reaction was mixed with 200 μl of the essential oil (400 mg) or standard (BHT, 400 mg) dilutions and 300 μl were dispensed by triplicate in a microplate for analysis. The absorbance was immediately (t = 0) measured at 470 nm in a Eon™Microplate Spectrophotometer (bioTek, VT, USA) against a blank, consisting of an emulsion only with β-carotene. Afterwards, the emulsion was incubated for 120 min at 45 °C, and the absorbance was recorded every twenty minutes.

#### Detection of apoptosis using flow cytometry

HaCaT cells (2.5 × 10^5^) were seeded in 24-well plates and treated with 1 µL of the EO of *C. sinensis* (0.842 µg) or *C. latifolia* (0.872 µg) with 250 µM of H_2_O_2_ for 24 h; afterwards, cultures were dissociated with 0.1% trypsin/EDTA in PBS 1X. The cells were then washed in PBS 1X and resuspended in binding buffer, and the apoptotic cell death rate was examined using Annexin V-APC and 7AAD double staining (incubation with 5 µl Annexin V-APC and 10 µl 7AAD for 15 min in the dark), according to the manufacturer’s protocol. Subsequently, the cells stained with Annexin V-APC/7-AAD were detected using a BD Accury C6 flow cytometer (BD Biosciences, CA, USA). Data acquisition and analyses were performed using Flowjo v10.3 software. Three independent biological replicates were performed by triplicate.

#### Measurement of intracellular reactive oxygen species

DHE (150 mM) and 1 µL of the EOs of *C. sinensis* (0.842 µg) or *C. latifolia* (0.872 µg) was added to HaCaT cell (2.5 × 10^5^ in 24-well plates), which were then incubated at 37 °C for 30 min in the dark, to determine the intracellular •O^2−^ concentrations. Samples were treated with 250 µM H_2_O_2_ to create oxidative stress. Cells were then washed, resuspended in PBS 1X, and maintained on ice for immediate detection by flow cytometry (BD Accury C6, CA, USA). Data were analyzed using the Flowjo v10.3 software. For fluorescence quantification samples were acquired in triplicate, and 10,000 events were used for each measurement. Cells were excited at 488 nm, and DHE fluorescence was detected using 585/42 (DHE) bandpass filters. Data were expressed as the geometric mean fluorescence intensity (MFI).

### Statistical analysis

Statistical analysis was performed using Bonferroni correction of a two-way ANOVA test using Graph-Pad Prism 5.0 software.
